# Artificial selection optimizes clonality in chaya (*Cnidoscolus aconitifolius*)

**DOI:** 10.1038/s41598-021-00592-0

**Published:** 2021-10-25

**Authors:** Miguel A. Munguía-Rosas

**Affiliations:** Laboratorio de Ecología Terrestre, Departamento de Ecología Humana, Centro de Investigación y de Estudios Avanzados del Instituto Politécnico Nacional (Cinvestav), 97310 Mérida, México

**Keywords:** Plant domestication, Plant ecology, Plant reproduction

## Abstract

The clonal propagation of crops offers several advantages to growers, such as skipping the juvenile phase, faster growth, and reduced mortality. However, it is not known if the wild ancestors of most clonal crops have a similar ability to reproduce clonally. Therefore, it is unclear whether clonality was an ancestral condition, or if it evolved during domestication in the majority of these crops. Here, I assessed some traits that are relevant to clonal propagation using stem cuttings from chaya (*Cnidoscolus aconitifolius*) and compared these traits to those of its wild ancestor. Chaya is highly relevant crop to food security in its domestication center (Yucatan Peninsula) and is now cultivated in several countries. Chaya is also an excellent model for assessing the effect of domestication on clonality because wild relatives and selection targets are known. Specifically, I compared resistance to desiccation, water and resource storage, as well as the production of new organs (shoots and leaves) by the stems of wild and domesticated plants. I also compared their performance in root development and clone survival. I found that, relative to their wild ancestors, the stem cuttings of domesticated chaya had 1.1 times greater storage capacity for water and starch. Additionally, the stems of domesticated plants produced 1.25 times more roots, 2.69 times more shoots and 1.94 more leaves, and their clones lived 1.87 times longer than their wild relatives. In conclusion, the results suggest that artificial selection has optimized water and starch storage by stems in chaya. Because these traits also confer greater fitness (i.e. increased fecundity and survival of clones), they can be considered adaptations to clonal propagation in the agroecosystems where this crop is cultivated.

## Introduction

Clonality is a term used to describe asexual reproduction by an individual resulting in a set of genetically identical descendants (except for the appearance of somatic mutations) or clones^1,2^. In angiosperms this can be achieved through apomictic seeds (i.e. seeds produced by unfertilized ovules) or vegetative organs, with the latter by far the most common in this group of plants^3^. Clonality is a derived trait among angiosperms and has evolved many times^4,5^. Clonal plants are common in wet, nutrient-poor, cold and shaded habitats^3,4^ where the conditions for sexual reproduction (e.g. scarce pollen vectors or mates), germination and establishment of sexual seeds and seedlings may be unfavorable^3^. The majority of clonal plants, if not all, are perennials with relatively reduced resource allocation to sexual organs^6–8^. The occurrence of clonality in phylogenetically unrelated species under a variety of ecological conditions suggests that this reproductive system can be selected for in some enviroments^3,4,6^.

Clonality is also common in crops. Clonal crops belong to 34 botanical families, are perennials and exhibit a wide range of life forms (trees, shrubs, herbs, and vines)^9,10^. A relevant proportion of clonal crops were domesticated in the wet tropics where today some species continue to coexist alongside their wild ancestors^11^. Because clonal propagation has brought several advantages to growers relative to sexual propagation (i.e. easier cultivation, greater survival and reduced time to reach sexual maturity)^9^ and some of the few clonal crops studied so far have wild ancestors that mainly reproduced sexually^12,13^, it is reasonable to think that clonality may also be a derived trait in some crops and that traits that facilitate clonal propagation have been artificially selected during the domestication process^9^. Clonality in crops may be also the result of selection for a different plant trait. For example, in some fruit trees, growers have selected for self-compatibility or parthenocarpy which allows trees to set fruit when pollinators or mates are scarce or absent. However, some of these trees are unable to reproduce sexually owing to the poor performance of selfed progeny or the absence of seeds; therefore, are clonally propagated^14,15^.

Using a comparative approach, some authors have suggested that vegetative organs used as propagules in clonal wild plants have undergone several physical (e.g. resistance to desiccation), anatomical (e.g. increased thickness) and physiological (e.g. resource storage) modifications that presumably maximized their efficiency as an organ for clonal propagation^5^. This may be also the case with clonal crops; however, such a complex evolutionary transition is hardly likely to have resulted from a “single event” domestication process—as previously postulated for these crops—that essentially consisted of cloning the wild plants that exhibited the desired phenotype^11^. More recently, the domestication of clonal crops has been recognized as a far more complex process^9,11^. The wild relatives of some clonal crops likely were managed in situ (i.e. incipient management of naturally recruited plants without transplantation)^16^ before being domesticated and, once brought into agroecosystems, their local adaptation to this new habitat was facilitated by humans when they selected genotypes that performed better^11,17^. Also, as modular organisms, a single plant produces multiple copies of the same organ that vary in age, size and nutrient content^16^, and each copy of a given organ may develop different somatic mutations that are susceptible to selection^19^. Therefore, in clonal crops, artificial selection may operate at the level of genotype (i.e. genetically different plants), ramet (i.e. plants with the same genotype or clones) or organ (e.g. branches)^9,20,21^. If the traits exhibited by the vegetative organs that were selected by growers affect their performance as propagules, this function is expected to be optimized by artificial selection^20,22^.

The claim that the vegetative organs of clonal crops, such as stems and roots, have been optimized to serve as propagules through artificial selection and that selected traits represent adaptations to clonality, is common in the literature^9,11,17,20,23,24^. However, there is little empirical evidence behind this assertion. This is probably because our knowledge about the reproductive biology of wild ancestors for most clonal crops is quite limited^11,13^. Given that the ancestral states of traits seen in domesticated plants are those exhibited by their closest wild relatives, it is virtually impossible to identify which traits of the vegetative organs used for clonal propagation have been optimized through domestication and to what extent, if the state of such traits is unknown in wild relatives^13^. Much of what we have learned on this topic comes from a series of studies performed on cassava (*Manihot esculenta*) and its closest wild relatives (*M. esculenta flabellifolia*)^9,13^. Relative to its wild progenitors, the stems of domesticated cassava exhibited a greater starch content, which may improve the performance of stems as organs for clonal propagation^13,21^. Larger stem cuttings are often selected by cassava growers to be used as propagules and this trait is a reliable predictor of the production of new shoots, roots and the yield (fresh mass of starchy roots) of cassava clones^20^. However, it is not known whether this was the result of domestication because the study was not replicated with the wild relatives^20^. Other authors have suggested that increased resistance to desiccation may be an adaptation of the stems of cultivated cassava to clonal propagation^13,25,26^. However, this had not been properly demonstrated. Thus, while some traits have been identified as potentially important for clonal propagation (e.g. resource storage, resistance to desiccation, production of new organs) and some of these traits are correlated with traits of interest to growers (i.e. stem size) ^9,13,20,22^, it is unclear whether these have been optimized by artificial selection (i.e. traits are greater in crops than in their wild progenitors) or whether they represent adaptations to clonality (i.e. lead to greater fitness). Since previous research has focused on cassava, it is also important to study other clonal crops to assess the generalizability of the results for cassava.

In this study, I used chaya (*Cnidoscolus aconitifolius*) as a study model to investigate how clonality has been optimized through domestication. Chaya is a vegetable crop (i.e. leaves are the edible organ) that was domesticated by the Maya on the Yucatan Peninsula^27,28^. While it is mainly grown in Mesoamerica, it has recently expanded to several areas of the world far beyond its native distribution range (e.g. some dry tropical regions in Africa and Asia)^29,30^. The closest wild relatives belong to the same species as the cultivar and they coexist in the centre of domestication^31,32^. In contrast to wild relatives that only reproduce sexually in nature, the cultivar rarely produces pollen or viable seeds, so chaya is clonally propagated using stem cuttings^12,32^. Selection targets are known, people select thicker stems from secondary branches to propagate plants with more and bigger leaves and with fewer trichomes^32,33^.

The objective of this study was to assess the major changes that vegetative organs (stems) have undergone during domestication and that maximize their novel function as propagules in chaya. I specifically asked the following questions: What are the changes that the chaya stems have undergone through domestication? Are these changes associated with their performance as propagules? And, have these traits been optimized through domestication; if so, to what extent? I predicted that domestication had optimized clonal propagation in chaya and therefore, the stem cuttings of domesticated plants would have traits associated with greater resource storage capacity and resistance to desiccation, and a greater capacity to produce new organs, as well as greater fitness relative to its wild relatives.

## Results

### Resistance to desiccation

**Figure 1:**
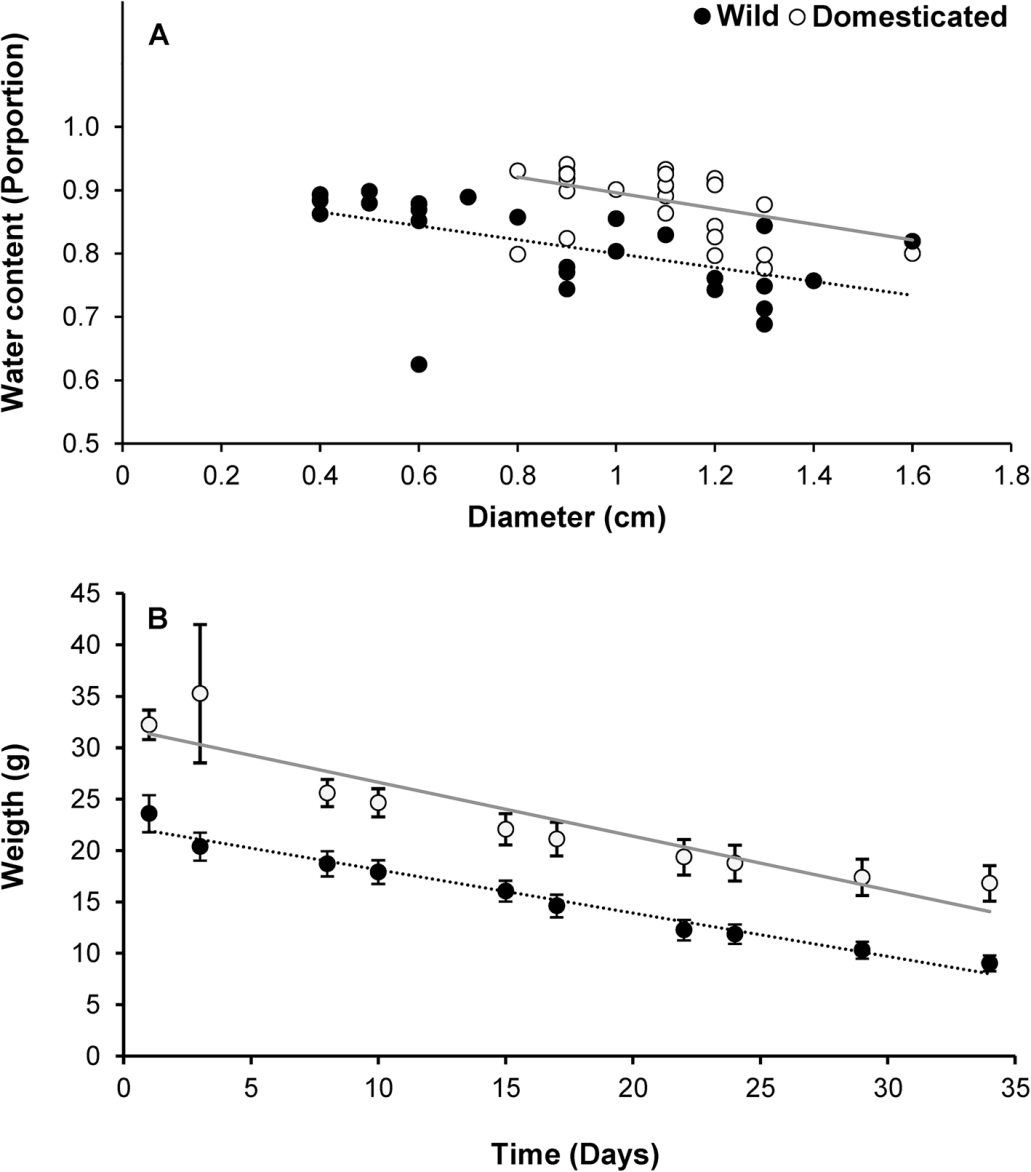
Water content (**A**) and weight loss (**B**) in stem cuttings of wild and domesticated *Cnidoscolus aconitifolius*. Circles in A correspond to individual cuttings and in (**B**), correspond to the mean (± 1 SE) of a sample of cuttings. The X axis in (**A**) shows the diameter of cuttings and the time elapsed since the stems were cut in (**B**). Gray regression lines correspond to the stems of domesticated plants and black dotted lines correspond to those of wild plants. Water content was assessed by weight differences in a cutting after being oven dried for 72 h, and weight loss was measured as the weight of a cutting twice a week under constant environmental conditions (26 °C, 12 h dark/light).

The stem cuttings of domesticated plants (88.87 ± 0.99%) had a greater water content than those of the wild plants did (81.23 ± 1.42%) (*F*_1,52_ = 24.91, *P* ≪ 0.01; hereafter comparison wild versus domesticated will be referred to as the domestication factor). Water content decreased slightly with the diameter of the cutting (coefficient: − 0.12 ± 0.5; *F*_1,52_ = 15.79, *P* ≪ 0.01), however, the interaction between the cutting’s diameter and domestication was not significant (*F*_1,52_ = 0.11, *P* = 0.74) (Fig. 1A). Stem cuttings of domesticated plants (32.21 ± 1.43 g) were also significantly heavier than those of the wild plants (23.58 ± 1.79 g; *F*_1,56_ = 21.04, *P* ≪ 0.01). Weight loss in stem cuttings was linear (coefficient: − 0.52 ± 0.08, *F*_1,512_ = 67.21, *P* ≪ 0.01); and the cuttings of wild and domesticated plants lost weight at similar rates (i.e. non-significant time x domestication interaction: *F*_1,512_ = 0.98, *P* = 0.32) (Fig. 1B).

### Resource storage

**Figure 2:**
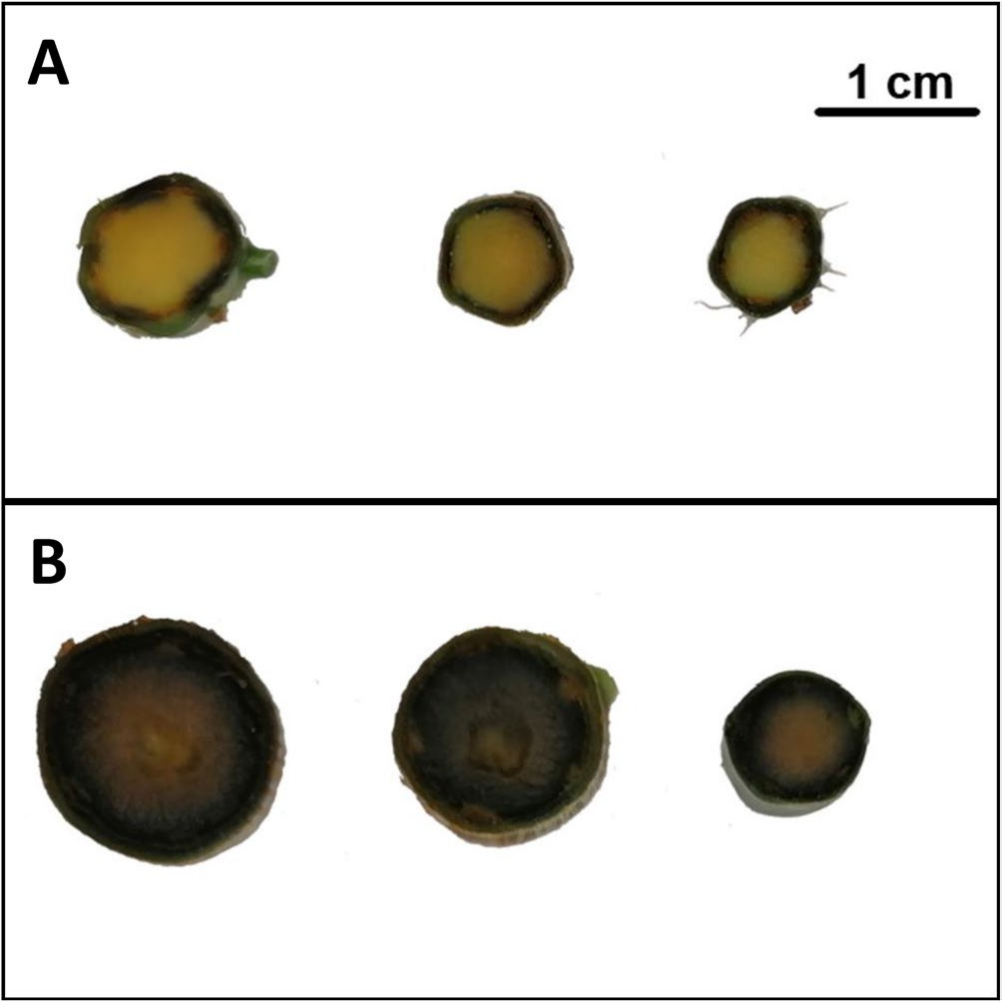
Transverse view of the stems of wild (**A**) and domesticated (**B**) plants stained with Lugol’s solution. Dark-blue areas indicate the presence of starch. The three stem sections show the variation in terms of size and staining patterns observed within the sample of wild (**A**) and domesticated plants (**B**).

Total soluble sugars were very similar in the stems of wild (4.62 ± 0.21%) and domesticated (4.71 ± 0.18%) plants. Domestication (*F*_1,36_ = 0.09, *P* = 0.78), stem diameter (*F*_1,36_ = 1.39, *P* = 0.24) and their interaction (*F*_1,36_ = 0.95, *P* = 0.33) had no effect on the percentage of total soluble sugars in the stems. Only a thin ring of starch was detected in the most external parts of cross-sections of the wild plant stems (Fig. 2A), but starch almost completely covered the area of stem sections in domesticated plants (Fig. 2B).

### Shoot production and cutting longevity

The vast majority of stem cuttings of domesticated plants (89.28%) developed at least one shoot during the study. In contrast, only one third of the cuttings from wild plants developed at least one shoot (Table 1). The incidence of shoots was statistically different between wild and domesticated plants (χ_1_^2^ = 19.85, *P* ≪ 0.01); however, it was not affected by the diameter (χ_1_^2^ = 2.93, *P* = 0.11) or the weight (χ_1_^2^ = 0.59, *P* = 0.44) of the cuttings at the beginning of the experiment. The number of shoots per cutting was 2.69 times greater in domesticated plants than in the wild plants (χ_1_^2^ = 19.85, *P* ≪ 0.01) (Table 1). As occurred with shoot incidence, neither the initial diameter (χ_1_^2^ = 0.37, *P* = 0.51) nor the weight (χ_1_^2^ = 1.71, *P* = 0.17) of stem cuttings significantly affected the number of shoots per cutting.

**Table 1:** Shoot incidence, number (count) and time (days) elapsed after the first shoot was observed in stem cuttings of wild (Wild) and domesticated (Domesticated) chaya (*Cnidoscolus aconitifolius*).

Trait	Wild	Domesticated
Shoot incidence	0.33^a^	0.89^b^
Number of shoots per cutting	0.40 ± 0.11^a^	2.89 ± 0.41^b^
Days elapsed until first shoot	13.54 ± 1.76^a^	9.08 ± 0.75^b^

Stems were kept under controlled environmental conditions (26 °C, 12 h light/dark) and had no external source of water or nutrients. Data are the mean ± 1 standard error, except for shoot incidence, for which the proportion of cuttings with at least one shoot at the end of the experiment is shown. Different superscript letters indicate statistically significant differences between the cuttings of wild and domesticated plants.

**Figure 3:**
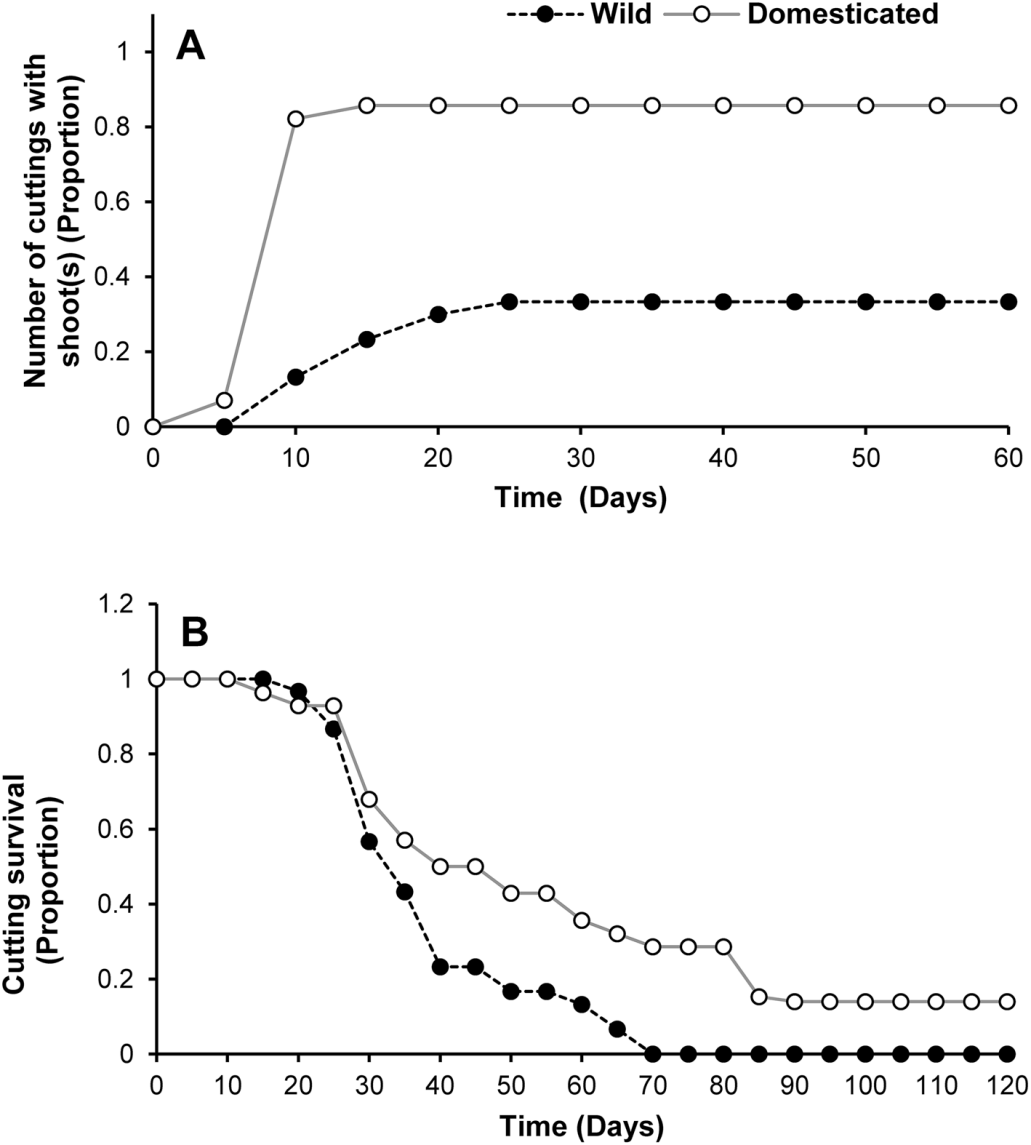
Cumulative proportion of cuttings of wild (Wild) and domesticated (Domesticated) plants of *Cnidoscolus aconitifolius* with at least one shoot (**A**). Survival curves of stem cuttings for wild and domesticated *C. aconitifolius* after 120 days of monitoring under controlled environmental conditions (**B**).

On average, the first shoot was observed ca. 4 days earlier in the cuttings of domesticated plants than in those from the wild (Z = 5.18, *P* ≪ 0.01) (Table 1; Fig. 3A). The highest proportion of cuttings with at least one shoot was reached in 15 and 25 days in the stem cuttings of domesticated and wild plants, respectively (Fig. 3A). The time the first shoot was observed was positively (coefficient = 0.08 ± 0.03) influenced by initial weight (Z = 2.52, *P* = 0.01) but negatively influenced (coefficient = − 2.52 ± 0.91) by the initial diameter of the cuttings (Z = − 2.74, *P* ≪ 0.01). Seventy days after the experiment began, all the cuttings from the wild plants had died. In contrast, four of 28 cuttings (survivorship = 0.14, 95% CI 0.06–0.35) from the domesticated plants were still alive after 120 days, when the experiment ended. Mean time to death was 36.63 ± 2.65 and 54.53 ± 6.22 days for cuttings from wild and domesticated plants, respectively. The survivorship curves for the cuttings of wild and domesticated plants were significantly different (Z = − 2.88, *P* ≪ 0.01) (Fig. 3B). Neither initial diameter (Z = − 1.23, *P* = 0.07) nor initial weight had a significant effect on the longevity of the cuttings (Z = 1.76, *P* = 0.08).

**Figure 4:**
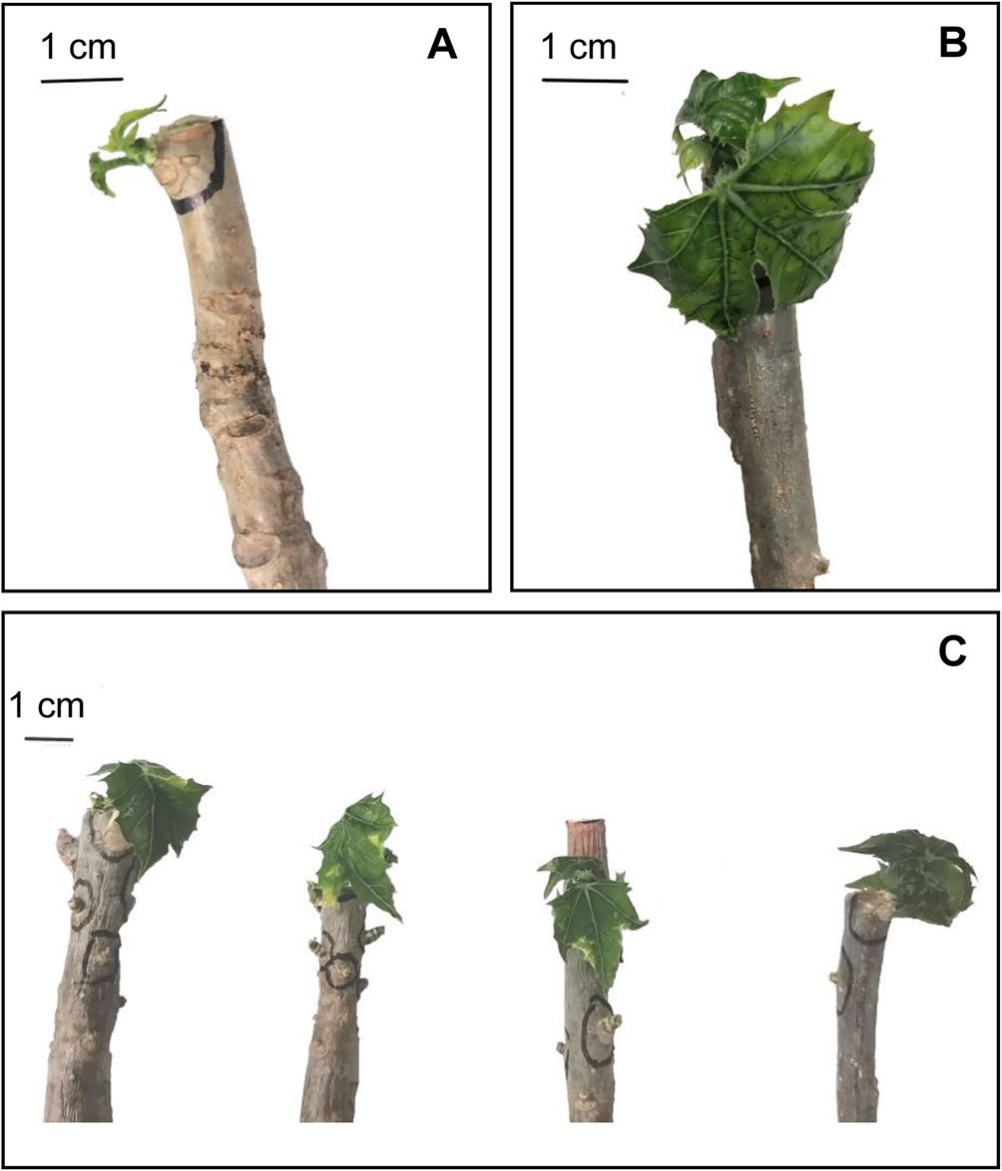
Stem cuttings from a wild (**A**) and a domesticated (**B**) *Cnidoscolus aconitifolius* 6 weeks after having been cut. Although the cuttings in (**A)** and (**B**) are the same age, the differences in terms of the degree of development of leaves is noteworthy. (**C**) Surviving cuttings of domesticated plants after 120 days, all of which were obtained from domesticated plants and were completely deprived of substrate and water. Scale bars represent 1 cm in all cases.

In contrast to the cuttings of wild plants, which produced only poorly developed leaves if any (Fig. 4A), most shoots from the cuttings of domesticated plants developed fully expanded leaves (Fig. 4B). After 120 days, when the experiment ended, the domesticated plant cuttings that survived also had vigorous, fully expanded leaves, and exhibited only minor signs of wilting (Fig. 4C).

### Rooting

**Figure 5:**
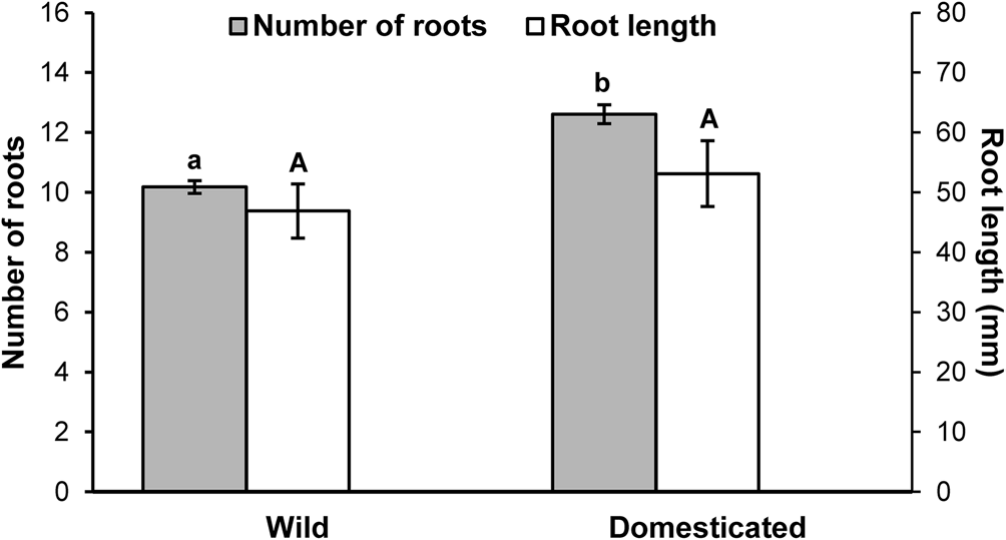
Number of main roots (number of roots) and length of the longest root (root length) of stem cuttings of wild and domesticated *Cnidoscolus aconitifolius*. Roots were counted and measured 1 month after being planted. Data are the mean ± 1 SE. Different letters indicate statistically significant differences between cuttings from wild and domesticated plants. Lower case letters correspond to the number of roots and upper case letters correspond to the length of the longest root.

One month after being planted, stem cuttings had a rooting incidence of 83% (wild) and 76% (domesticated), however, this difference was not statistically significant (χ_1_^2^ = 0.41, *P* = 0.52). All the cuttings from wild plants that did not develop roots showed severe signs of rotting, but in only one third of the cuttings from domesticated plants that did not develop roots, was there rotting, which was minor, and those cuttings did develop leaves. Cuttings from domesticated plants (12.61 ± 0.32) produced 1.24 times more roots than the cuttings of wild plants did (10.18 ± 0.21) and this difference was statistically significant (χ_1_^2^ = 5.81, *P* = 0.02) (Fig. 5). The number of roots was also positively (coefficient = 0.68 ± 0.21) and significantly (χ_1_^2^ = 19.09, *P* ≪ 0.01) affected by the initial diameter of cuttings, but the interaction between this covariable and domestication was not statistically significant (χ_1_^2^ = 2.51, *P* = 0.11). The length of the longest root was not statistically different (*F*_1,41_ = 0.74, *P* = 0.39) between cuttings from wild (46.91 ± 4.52) and domesticated plants (53.13 ± 5.52) (Fig. 5). Similarly, neither the effects of initial diameter (*F*_1,41_ = 0.22, *P* = 0.63) nor its interaction with domestication (*F*_1,41_ = 1.04, *P* = 0.31) on root length were statistically significant.

While all rooted cuttings from domesticated plants, except one, developed at least one leaf (97%), only 50% of rooted cuttings from wild plants did (χ_1_^2^ = 9.76, *P* ≪ 0.01). However, neither the number of roots (*Z* = 1.64, *P* = 0.12) nor the length of the main root (*Z* = 1.81, *P* = 0.07) were reliable predictors of the presence/absence of leaves in the cuttings from the wild plants.

### Clone survival

**Figure 6:**
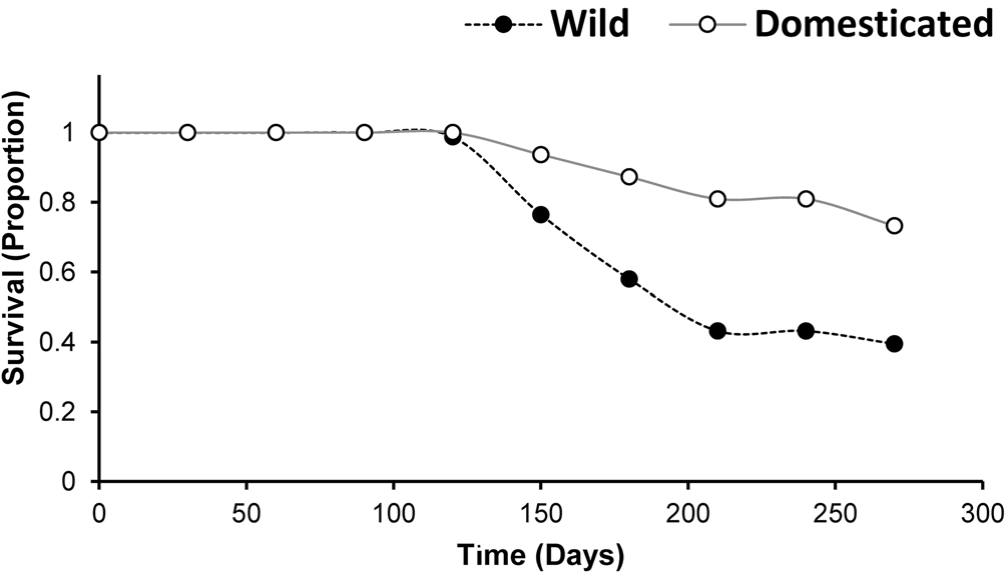
Survival of clones propagated using stem cuttings of wild and domesticated *Cnidoscolus aconitifolius*. Plants were maintained in a plant nursery in a common garden for 9 months (270 days).

During the experiment, 21 (26.58%) and 49 (60.49%) of the clones from domesticated and wild plants died, respectively. Mean time to death was 211.31 ± 10.06 and 182.41 ± 5.06 days for domesticated and wild clones. At the end of this period, survival was 0.73 (95% CI 0.64–0.84) for clones from domesticated plants and 0.39 (95% CI 0.31–0.52) for wild plant clones. Survival curves for the clones of wild and domesticated plants were significantly different (Z = − 3.75, *P* ≪ 0.01) (Fig. 6).

## Discussion

In this study I have shown that the stems of domesticated chaya, a clonally propagated crop, have a greater capacity to store water and starch than do those of its wild relatives. Once planted, the aerial (greater and faster shoot production and longevity) and the subterranean (greater number of roots) parts of stem cuttings also performed better in the cultivar than in its wild ancestors. Moreover, the cultivar clones had greater survivorship than did their wild relatives when grown in a common garden. Observed differences in the traits of stems of wild and domesticated plants and in their performance when used as propagules were likely the result of artificial selection, consciously and unconsciously carried out by humans during the domestication of chaya. Since the traits that are selected for also increased the fecundity (sensu Elias et al.^20^) and survivorship of cultivated chaya, they can be considered adaptations to clonal propagation in human-created habitats^34^.

Resistance to desiccation has been mentioned as an artificially-selected trait that may have contributed to improving the performance of stems as propagules in clonal crops^13,25,26^. However, this was not the case for chaya because stem cuttings lose water at the same rate in both wild and domesticated plants. While the stem cuttings of wild and domesticated plants had the same resistance to desiccation, the greater water content observed in the cuttings of domesticated plants may delay dehydration relative to that which occurs in wild plant cuttings under similar environmental conditions.

A notable difference in the stems from wild and domesticated plants was starch storage. Starch almost completely covered the internal part of the stems of domesticated chaya, but was restricted to a small area in the most external part of stems in its wild relatives. Starch is accumulated in the sink organs (typically roots and stems) of some plant species, and this is considered a strategy to maintain growth under unpredictable adverse environmental conditions or to restart vegetative growth when adverse conditions are alleviated in seasonal environments^35^. Molecular evidence suggests that the starch content of sink organs can also be maximized by means of artificial selection in other clonal crops (e.g. cassava^36^, potato^37^, yam^38^). A high concentration of starch in stems and/or roots is highly desirable in some clonal crops (e.g. sugar cane, potato, cassava), where its presence and concentration is easily detected by humans through the sense of taste and thus, humans consciously have selected for a high starch content^11,20^, however, the stems of chaya are not edible. It is likely that starch content in chaya is correlated with a visible, correlated trait selected for by growers, such as stem thickness. However, this correlation was not tested in this study because I measured starch content qualitatively (i.e. presence/absence, and distribution inside cuttings). Therefore, future research should quantify the starch content in cuttings of chaya in greater depth to determine whether it is correlated with stem thickness. If the thicker cuttings selected by growers have also more starch, the cuttings may be able to mobilize these carbohydrates to generate new organs. The greater content of starch observed in domesticated plants could be the result of selection for plants with greater leaf production^32^ which may be also more prone to producing an excess of photosynthates under the benign environmental conditions that prevail in agroecosystems and may store them as starches in sink organs like stems^39,40^. Also, further research is needed to identify in greater detail the physiological/biochemical mechanism(s) that underlie the greater starch storage observed in the stems of domesticated chaya.

Unrooted cuttings were able to produce shoots and leaves, however, the cultivar’s cuttings performed far better than those of the wild plants did. Not only did most cuttings produce shoots but they also produced more, earlier and at a faster rate. Although the longevity of cultivar cuttings was 1.5 times greater than that of the wild cuttings, among-cutting variation was notable. The unrooted cuttings of wild plants survived as long as 2 months, but amazingly, through artificial selection, the longevity of cuttings was double that. For logistical reasons, I had to conclude the experiment after 4 months, however, four cultivar cuttings were still alive at the end of the experiment. Therefore, the maximum longevity of these cuttings is unknown but is longer than 4 months. This extraordinary longevity of unrooted cuttings is long enough to survive the entire drought season in the study area, and is similar to that of other clonal plants with specialized subterranean storage organs^40^. Because the cuttings were unrooted and had no source of water or nutrients, the most obvious explanation for the success of the domesticated cuttings, in terms of shoot production and longevity, is their greater internal content of starch and water.

The cuttings of both wild and domesticated chaya have a great (ca. 80%) chance of rooting and develop roots of similar length when planted. There is little doubt that this pre-existing condition facilitated the domestication of chaya. The existence of hard-to-root cuttings, even in intensively managed modern crops, suggests that artificial selection has not always led to the optimization of rooting^41,42^. On the other hand, the number of roots was greater in the cultivar than in its wild relatives, suggesting that artificial selection has optimized this trait. Again, I suggest that the greater amount of endogenous starch observed in cultivated chaya may explain this result. Agronomic studies have also shown that endogenous sugar positively affects rooting in the cuttings of perennial woody crops^43^, supporting this idea. Root traits are usually invisible organs for growers; however, previous studies have shown that the traits consciously selected by growers, such as thicker and larger stems, are good predictors of rooting in cassava^20^. This seems to be the case for chaya, in which growers also select thicker stems as a source of cuttings^32^ and, as reported in this study, thicker cuttings also produce more roots. An interesting issue to be explored is what contribution the leaves produced prior root development (a condition more frequently seen in the cultivar) may make to the energetic budget and the performance of the cuttings. Cuttings of the cultivar not only produced more roots, but also rooted clones, with nearly two times greater survivorship than that of its wild counterpart during the first 9 months. Thus, when clonally propagated, cultivated chaya has a greater chance of survival in human-created environments than their wild relatives do. The greater survivorship observed in clones of domesticated plants could be an effect of the greater internal resources and water in the propagules, as well as their greater efficiency in producing roots, shoots and leaves. Rooting success and clone survival are highly relevant in the context of artificial selection because these are considered components of fitness in clonal plants^20,34^.

One issue that needs to be acknowledged is the localities where plant accessions were collected to establish the experimental orchard used as a source of cuttings for this study. These localities only represented a subset of the whole distribution of *C. aconitifolius*. Therefore, natural variation range of the traits measured may have been underestimated in the present study^30^. While I cannot completely rule out the existence of unsampled wild plants with a similar (or superior) ability to propagate clonally, I think that this probability is low. The clonal propagation ability exhibited by domesticated plants is extraordinary and a wild plant with this ability would probably become the dominant phenotype quite rapidly, at least in some localities. However, I have never seen any fallen branch develop new shoots or leaves in natural habitats during extensive field work throughout chaya’s center of domestication.

In conclusion, I have found strong evidence that artificial selection has optimized chaya stems for clonal propagation. Relative to their wild ancestors, the stems of domesticated chaya have a greater capacity for water and carbohydrate storage. As these stem traits are linked to greater fecundity and clone survival, they can be considered adaptations. In contrast to seeds, the use of vegetative propagules skips the juvenile phase, allows for rapid growth, and reduces mortality^44,45^. These advantages can easily compensate for some of the associated disadvantages, such as greater desiccation and limited dispersal when grown in agroecosystems tended by humans. These results represent an important advance in our understanding of the evolution under domestication of clonality. In contrast to the traditional view that the domestication of clonal plants was a one-step event^11,17,24^, in this study I have shown that a single organ may have undergone several human-driven changes in its anatomy, physiological processes and resource allocation patterns. My findings are in line with the more recent view that the domestication of clonal crops has been a process of adaptation in which numerous selection cycles (i.e. generations) were probably required^9,13^. The transition from a purely vegetative function to vegetative and reproductive functions in modified stems (stolons, rhizomes, cladodes) has evolved several times in wild angiosperms^5^, therefore, clonal crops and their wild relatives may also help us understand this process during early species divergence^8,13^.

## Methods

### Study species

*Cnidoscolus aconitifolius* (Euphorbiaceae) is a shrub that grows up to 5 m tall, native to Mexico and Central America^30,46^. The domesticated form is called “chaya” and is cultivated for its leaves^27,28^. Domestication syndrome includes the increased production of bigger leaves with significantly fewer trichomes and more succulent stems^32,33^. The chaya cultivar is clonally propagated from stem cuttings, and traditional growers usually select the thicker stems of secondary branches from apparently healthy plants for propagation^27,32^. Wild and domesticated plants may coexist, however, there is almost complete reproductive isolation between them due to poor pollen production by the cultivar^12^. *C. aconitifolius* is tetraploid (n = 18, x = 9), a condition that likely emerged prior to domestication because most wild relatives of the genus studied so far (*C. multilobus*, *C. rotudifolius*, *C. stimulosus*, *C. tubulosus*, *C. urens*) are also tetraploid^47,48^.

This study complied with the relevant institutional, national, and international guidelines and legislation. *Cnidoscolus aconitifolius* is not at risk of extinction or under the protection of international or local authorities. Additionally, the plant material (stem cuttings) used in this study was collected from private land and therefore no permission from the local government was required. The identity of a specimen was confirmed by the curator (Dr. J. Tun-Garrido) of the Alfredo Barrera Marín herbarium where a specimen was deposited (Voucher: UADY-23474).

### Resistance to desiccation

As a part of a bigger project, in summer 2007, I cut two to four stems from 60 plants (30 wild and 30 domesticated) from the secondary branches of adult plants in 40 sites scattered all over the Yucatan peninsula (details in reference^32^). Wild and domesticated plants were easily differentiated in the field because the stems of wild plants clearly have more trichomes than domesticated plants do. Also, all domesticated plants were collected in home gardens and wild plants were collected from nearby secondary forests. All three known cultivated varieties of chaya present in the study area were sampled^27–29^. Immediately after collection, I planted stem cuttings in an experimental orchard in the municipality of Merida in central Yucatan. In autumn 2020, when this study started, only 60 plants (30 wild and 30 domesticated) had survived; all were sexually mature, the same age and similar in height (2–2.5 m tall). All of the plants in the orchard were exposed to full sunlight and watered evenly once a week. I took 60 stem cuttings (35 cm long) from the secondary branches of 60 different plants (30 wild and 30 domesticated) from the experimental orchard. I used this orchard as source of cuttings to keep the environment and mother plants as homogeneous as possible. Once in the laboratory, the basal diameter of the cuttings was measured, and a 5 cm segment was cut from the 35-cm-long segment. Both segments (30 cm & 5 cm) were weighed for all cuttings and the small ones were oven-dried at 75 °C for 72 h to estimate water content. The 30 cm segments were placed in a controlled environment chamber (Binder Inc., KBW 240, Tuttlingen, Germany) at a constant temperature of 26 °C with a photoperiod of 12 h light/dark, light provided by high-pressure sodium lamps (PFD = 46.89 µmol m^2^ s^−1^). The only source of water for the cuttings was environmental humidity (≈ 60%) which was homogeneous throughout the chamber. All cuttings were weighed twice a week for a month to estimate the rate of water loss (measured as weight loss). The temperature and photoperiod used resembled the average values observed during the summer on the Yucatan Peninsula.

### Resource storage

Using the same source plants, selection criteria and the sampling design outlined in the previous section, 1 month later, I cut 60, 10 cm-long stems from 60 different plants (30 wild and 30 domesticated) to measure soluble sugars and assess the presence of starch. To identify the presence and distribution of starch, I cut a fine slice (3–4 mm thick) from the end opposite the apex of each stem section and immediately added approximately 1 ml of Lugo’s solution (5 g I_2_ + 10 g KI + 85 ml H_2_O) to all the slices simultaneously. After 3 min, the slices were washed in distilled water to remove the excess solution. The presence and distribution of starch was easily recognized as it stains dark blue. I indirectly measured total soluble sugars in the remaining portion of the stems by using the method outlined by Okamura and colleagues^49^, which consists of measuring the sugar content of the sap, obtained by squeezing the stem sections, and using a digital refractometer with automatic temperature compensation (HI96801, Hanna Instruments Inc., Rhode Island, USA). Although the refractometer gives the sugar content in Brix units, it is a reliable proxy for % total soluble sugars (r = 0.96, *P* < 0.01)^47^. I was able to obtain enough sap to perform the measurements on the stems of 19 wild and 21 domesticated plants (one stem per plant); the remaining stems were too hard and/or too dry to obtain enough sap to test.

### Shoot production and cutting longevity

Twice a week over 4 months (120 days), I counted the new shoots sprouting from the 60, 30-cm-long cuttings (from 60 plants: 30 wild and 30 domesticated) in the controlled environment chamber described above (for sampling design see the *Resistance to desiccation* subsection). The presence of leaves emerging from the shoots was also recorded. During the experiment, all new shoots were labeled to avoid underestimating the total number of shoots because some of them withered and fell off during the experiment. In addition to shoot emergence time, I recorded the time to cutting death, defined as the time when a cutting showed clear signs of wilting, shoot abortion or leaf abscission (when these occurred). I discarded dead cuttings to prevent the proliferation of fungi and bacteria in the chamber, and the potential contamination of the remaining cuttings.

### Rooting

In January 2021, I took 57 stem cuttings, 30 cm in length, from 56 plants (26 wild and 30 domesticated) following the same procedure and sampling design described in the subsection *Resistance to desiccation*. Two days after collection, the cuttings were planted in 2L plastic pots using a mix of gravel and soil (70:30) as the substrate. Before planting the cuttings, I removed all of the leaves with pruning shears. I left the pots with these cuttings in a plant nursery located next to the experimental orchard mentioned before. All cuttings were exposed to the same light environment (full light exposure) and watered to field capacity once or twice a week during the experiment. After 4 weeks, I gently removed the cuttings by turning the pot upside down, without pulling on the cutting in order to prevent any damage to the roots. Once the cutting had been removed, I washed the part that had been buried to eliminate all traces of soil. I carefully examined the cuttings with a magnifying glass in search of roots. I recorded the presence/absence of roots, the number of main roots (i.e. roots that emerged directly from the cuttings) and measured the length of the longest root, if present. Additionally, the presence/absence of any leaves on the aerial part of all cuttings was recorded.

### Clone survival

To assess the long-term survivorship of clonally propagated plants (clones), in June 2019, 160 stem cuttings (30 cm long) from 40 different mother plants (20 wild and 20 domesticated, four cuttings per plant) were collected using the same donors and according to the procedure and sampling design described in the *Resistance to desiccation* subsection. The cuttings were planted in 20 L pots using the same substrate and procedure described in the *Rooting* subsection. The plants were left in the plant nursery described above, under full light exposure and were watered to field capacity once a week. I checked clone survivorship monthly for 9 months (270 days) starting after the fifth month. I did this because survivorship is difficult to assess during the first months of life. For example, cuttings may have no leaves, but may have roots, or may even already have died with no clear signs of wilting because watering keeps the aerial part of the cuttings turgid. A clone was considered dead when it had lost its leaves and the stem presented clear signs of wilting.

### Statistical analyses

#### Resistance to desiccation

I estimated water content by subtracting the final weight of oven-dried cuttings from their initial weight. Water content (W), expressed as a proportion of total weight, was compared between the stems of wild and domesticated plants (i.e. domestication factor [D]) with an ANCOVA, including the diameter (DI) of fresh cuttings as a covariable. The domestication x diameter interaction was also included in the model. To improve the normality of the data, the proportion of water was arcsine square root transformed. The model fitted was: W_ijk_ = β + D_i_ + DI_j_ + (D DI)_ij_ + Ɛ_ijk_, where β was the intercept and Ɛ as the error term in this and models described below. The rate of water loss was assessed with a mixed-linear model, with weight as the response variable (WL) and domestication factor (D), time (T) and their interaction as fixed effects. The Cutting (C) term was also included as a block in the random part of the model to account for repeated measures. Therefore, the fitted model was: WL_ijkl_ = D_i_ + T_j_ + (D T)_ij_ + C_k_ + Ɛ_ijkl_.

The significance of the parameters in this and the models described below was assessed using Wald’s tests and the likelihood ratio test for random effects in mixed models.

#### Resource storage

I assessed differences in total soluble sugar (SG) between the stems of wild and domesticated plants (D) with an ANCOVA test, including stem diameter (DI) and its interaction with domestication factor in the model. The fitted model was: SG_ijk_ = β + D_i_ + DI_j_ + (D DI)_ij_ + Ɛ_ijk_.

#### Shoot production and cutting longevity

The effect of domestication (D) on shoot incidence (SI) and the total number of shoots (SN) was assessed using generalized linear models with a binomial (shoot sprouting incidence) and Poisson (number of shoots) error distribution. In both models, the initial diameter (DI) and weight (W) of the cuttings were included as covariables. The models fitted for the incidence and the number of shoots were: logit (SI_ijkl_) = β + D_i_ + DI_j_ + W_k_ + Ɛ_ijkl,_ and, log (SN_ijkl_) = β + D_i_ + DI_j_ + W_k_ + Ɛ_ijkl_.

The effect of domestication (D) on the time when the first shoot (t) was recorded and cutting survival (T) were assessed with time to an event (survival) analyses assuming an exponential distribution with constant hazard (h(t)). In both models, the initial diameter (DI) and weight (W) of cuttings were included as covariables. The fitted models were: h(t_ijk_) = h_0_(t_ijk_) exp (D_i_ + DI_j_ + W_k_) and h(T_ijk_) = h_0_(T_ijk_) exp (D_i_ + DI_j_ + W_k_).

#### Rooting

Rooting incidence (R), the number of roots (RN) and the length of the longest root (RL) were compared between the cuttings of wild and domesticated plants (D) using generalized lineal models (3 models in total) with binomial, Poisson and Gaussian error distributions, respectively. In all models, the initial diameter (DI) of the cuttings and its interaction with domestication were included as explanatory variables. The fitted models were: logit (R_ijk_) = β + D_i_ + D_ij_ + (D DI)_ij_ + Ɛ_ijk_, log (R_ijk_) = β + D_i_ + D_ij_ + (D DI)_ij_ + Ɛ_ijk_ and RL = β + D_i_ + D_ij_ + (D DI)_ij_ + Ɛ_ijk_.

The proportion of cuttings that developed leaves was compared between wild and domesticated plants with a proportion test. I assessed whether the number of main roots (RNW) and the length of the longest root (RLW) predicted the development of leaves (presence vs. absence of leaves) on cuttings from wild plants (LW) using a generalized linear model with a binomial error distribution. I did not include the data for the cuttings of domesticated plants in this analysis because all except one developed at least one leaf. The fitted model was: logit (LW_ijk_) = β + RNW_i_ + RLW_j_ + Ɛ_ijk_.

#### Clone survival

The survivorship of clones (tc) propagated from the cuttings of wild and domesticated plants were compared with survival models assuming a constant (exponential) hazard. The fitted model was: h(tc_i_) = h_0_(t_ci_) exp (D_i_). I did not include the identity of the mother in the model because I lost this data (labels were illegible due to rain and sun). However, I assumed that the variance explained by the mother plant was low because the phenotype of mother within each domestication level (wild vs. domesticated) was very similar.

All data analyses were run in R 0.6.2^50^.

## Supplementary Information

Supplementary Information.

## Data Availability

The raw data is included as online supplementary material.
